# A translational mechanistic synthesis of ischemia–reperfusion injury in experimental flap models toward free flap salvage

**DOI:** 10.3389/fsurg.2026.1824889

**Published:** 2026-04-20

**Authors:** Ovunc Akdemir

**Affiliations:** Department of Plastic, Reconstructive and Aesthetic Surgery, Faculty of Medicine, Istanbul Aydın University, Istanbul, Türkiye

**Keywords:** experimental flap models, free flap salvage, ischemia–reperfusion injury, microsurgery, microvascular dysfunction, oxidative stress, translational synthesis

## Abstract

**Background:**

Ischemia–reperfusion (I/R) injury remains a principal biological determinant of partial or total flap failure in reconstructive microsurgery. Reperfusion paradoxically initiates a coordinated cascade involving reactive oxygen species generation, lipid peroxidation, neutrophil activation, endothelial dysfunction, and microvascular obstruction, ultimately propagating progressive tissue necrosis. Despite extensive experimental investigation, effective translation into perioperative free flap salvage strategies remains limited.

**Methods:**

A structured translational synthesis was conducted integrating institutional experimental flap I/R studies performed over two decades with systematically mapped external literature published between 2000 and February 2026. Study identification followed PRISMA-informed search principles to ensure methodological transparency. Data extraction adhered to ARRIVE 2.0 domains to standardize experimental quality assessment. Given predefined biological heterogeneity in flap type, ischemia duration, intervention timing, and outcome definitions, quantitative meta-analysis was not pursued. Instead, biologically stratified comparative analyses were performed, and biologically contextualized viability changes were descriptively evaluated within comparable severe ischemia subgroups to preserve mechanistic interpretability.

**Results:**

Across experimental platforms, effective interventions demonstrated a reproducible biological signature characterized by attenuation of lipid peroxidation, suppression of neutrophil-mediated inflammation, restoration of endogenous antioxidant defenses, and preservation of nitric oxide bioavailability. In a comparable severe ischemia epigastric island flap paradigm, trimetazidine, propionyl-L-carnitine, and lutein each demonstrated improved survival area relative to ischemic controls within their respective experimental contexts. Surgical conditioning strategies exhibited robust protection, with venous flap pre-arterialization and delay procedures achieving survival rates approaching near-complete viability in the respective model. However, these conditioning strategies are not directly transferable to acute free flap salvage scenarios and are primarily applicable to planned or staged reconstructive settings.

**Conclusion:**

Flap I/R injury follows a reproducible oxidative stress–inflammation–microvascular dysfunction axis. Interventions targeting multiple components of this cascade appear to demonstrate a more reproducible protective pattern across severe ischemia conditions within their respective experimental contexts. These findings establish a translational mechanistic framework to guide rational adjunctive strategies in high-risk free flap protocols and support prospective clinical integration in microsurgical salvage scenarios. This synthesis is intended to guide mechanistic prioritization rather than imply direct interventional equivalence across models.

## Introduction

Ischemia–reperfusion (I/R) injury remains a decisive biological determinant of partial or total flap failure in reconstructive microsurgery. Although technical advances have increased free flap success rates, vascular compromise followed by reperfusion can precipitate a secondary injury that may exceed the primary ischemic insult. This “reperfusion paradox” is driven by a coordinated cascade including reactive oxygen species (ROS) generation, lipid peroxidation, neutrophil recruitment and myeloperoxidase (MPO) activation, endothelial dysfunction with nitric oxide (NO) imbalance, and microvascular obstruction (“no-reflow”), culminating in progressive tissue necrosis (1–3).

Experimental flap models consistently reproduce this oxidative stress–inflammation–microvascular dysfunction axis and have been widely used to test pharmacologic modulation and surgical conditioning strategies (4–6). Numerous antioxidant and metabolic agents—including taurine, melatonin, ceruloplasmin, trimetazidine, and propionyl-L-carnitine—have demonstrated varying degrees of protection against ischemia–reperfusion injury in experimental flap models by reducing oxidative stress, inflammatory infiltration, and tissue necrosis while improving microvascular perfusion and flap viability (7–11). However, translation into standardized perioperative adjuncts for free flap salvage remains limited—likely reflecting heterogeneity in flap type, ischemia severity, intervention timing, endpoint definitions, and measurement time points (2, 3).

**Figure 1:**
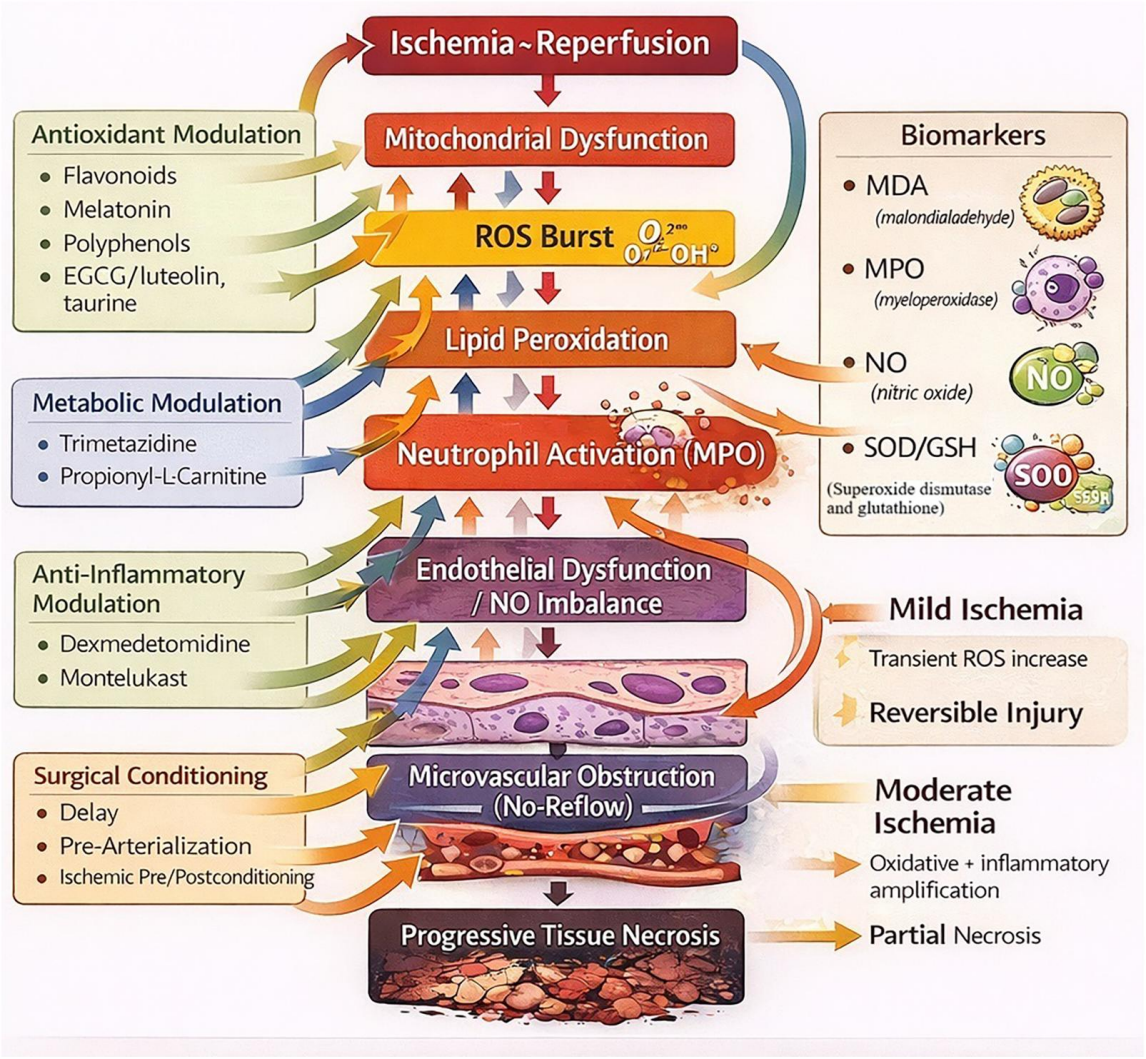
Mechanistic cascade of ischemia–reperfusion injury in flap surgery. Ischemia–reperfusion induces mitochondrial dysfunction and reactive oxygen species (ROS) generation, triggering lipid peroxidation, neutrophil activation (MPO), endothelial dysfunction with nitric oxide (NO) imbalance, and microvascular obstruction (no-reflow), ultimately leading to progressive tissue necrosis. Pharmacologic and surgical interventions act at different nodes of this oxidative stress–inflammation–microvascular dysfunction axis, converging on multi-level protection of flap viability. Arrows indicate bidirectional amplification loops between oxidative stress, inflammation, and endothelial dysfunction. This schematic figure was created with the assistance of AI-based visualization tools for graphical rendering only. All scientific content and conceptual design were developed by the authors.

Over the last two decades, our group has investigated both surgical conditioning and pharmacologic modulation across multiple flap paradigms (muscle, random-pattern skin, epigastric island, and venous flaps) using consistent mechanistic endpoints (7–13). The present work provides a structured translational synthesis integrating our institutional experimental series with a systematically mapped external literature (2000–February 2026), aiming to (i) identify reproducible mechanistic signatures linked to improved macro-viability, (ii) position negative findings as mechanistic clarification rather than weakness, and (iii) build a practical translational framework relevant to high-risk ischemia scenarios and free flap salvage. A total of 24 *in vivo* experimental investigations met predefined eligibility criteria and were integrated into this structured mechanistic synthesis (9 institutional, 15 external comparator studies). The mechanistic cascade of ischemia–reperfusion injury and the principal biological targets explored in these studies are illustrated in Figure 1. Importantly, this study does not aim to perform a quantitative comparison between interventions or flap models. Instead, the objective is to identify reproducible mechanistic patterns across heterogeneous experimental systems and to generate a translational framework for hypothesis generation.

## Methods

### Study design

Structured translational synthesis (narrative review with mechanistic mapping). Only studies reporting both macroscopic viability outcomes and mechanistic biomarker endpoints were included to allow mechanistic mapping. This approach aligns with PRISMA-informed transparency principles but is not intended as a formal systematic review or meta-analysis.This study was designed as a structured narrative translational synthesis rather than a formal systematic review, as the primary aim was mechanistic integration rather than quantitative effect estimation (14, 15). Screening and eligibility assessment were independently performed by two reviewers, with disagreements resolved by consensus.

### Data sources and search strategy

PubMed/MEDLINE, Scopus, Web of Science, and Embase were screened for *in vivo* experimental flap I/R studies published between January 2000 and February 2026. Search strings combined flap model terms with I/R terms and intervention terms, for example:

The core search string included combinations of:

(“skin flap” OR “muscle flap” OR “epigastric island flap” OR “venous flap”) and (“ischemia reperfusion” OR “I/R injury”) and (rat OR experimental) and (antioxidant OR metabolic modulation OR pharmacologic OR conditioning).

Reference lists of key reviews and high-relevance experimental papers were hand-screened to identify additional eligible studies (14–16). Searches were performed using a combination of free-text terms and controlled vocabulary (MeSH where applicable). Title/Abstract fields were primarily used, and database-specific indexing variability was addressed by combining multiple synonymous terms. The search strategy was systematically applied across databases using predefined keyword combinations, with Boolean operators (AND/OR) used to optimize sensitivity and specificity of retrieval.

### Eligibility criteria

#### Inclusion

1. *In vivo* experimental flap model (cutaneous, muscle, island, venous).
2. Defined ischemia followed by reperfusion.
3. At least one macroscopic viability outcome (survival area/necrosis rate).
4. At least one mechanistic marker (e.g., MDA, MPO, GSH, SOD/CAT, NO, cytokines, neutrophil infiltration).
5. Sufficient methodological clarity to interpret effect directionality and timing.

#### Exclusion

- *In vitro*-only studies.
- Clinical case reports/series without experimental flap I/R model.
- Non-flap transplantation models.
- Studies without quantifiable endpoints.

Eligibility and reporting transparency followed principles recommended for systematic evidence synthesis reporting (14, 15).

### Data extraction and quality domain mapping

Data extraction followed ARRIVE 2.0 domains to standardize methodological reporting and interpretability: animal characteristics, randomization/blinding where stated, ischemia/reperfusion protocol, intervention timing/dose, endpoint timing, and outcome reporting (17).

This review was not registered in PROSPERO because it was designed as a structured narrative synthesis and no quantitative meta-analysis was planned *a priori*.

Records identified through database searching totaled 1,690 (PubMed *n* = 458, Scopus *n* = 541, Web of Science *n* = 691).

- After removal of evident duplicates (*n* = 640), 1,050 records underwent title and abstract screening.
- Following screening, 970 records were excluded for not meeting predefined eligibility criteria.
- Eighty full-text articles were assessed for eligibility. Fifty-six were excluded due to absence of a defined *in vivo* flap I/R model, lack of macroscopic viability outcomes, or insufficient mechanistic reporting.

**Figure 2:**
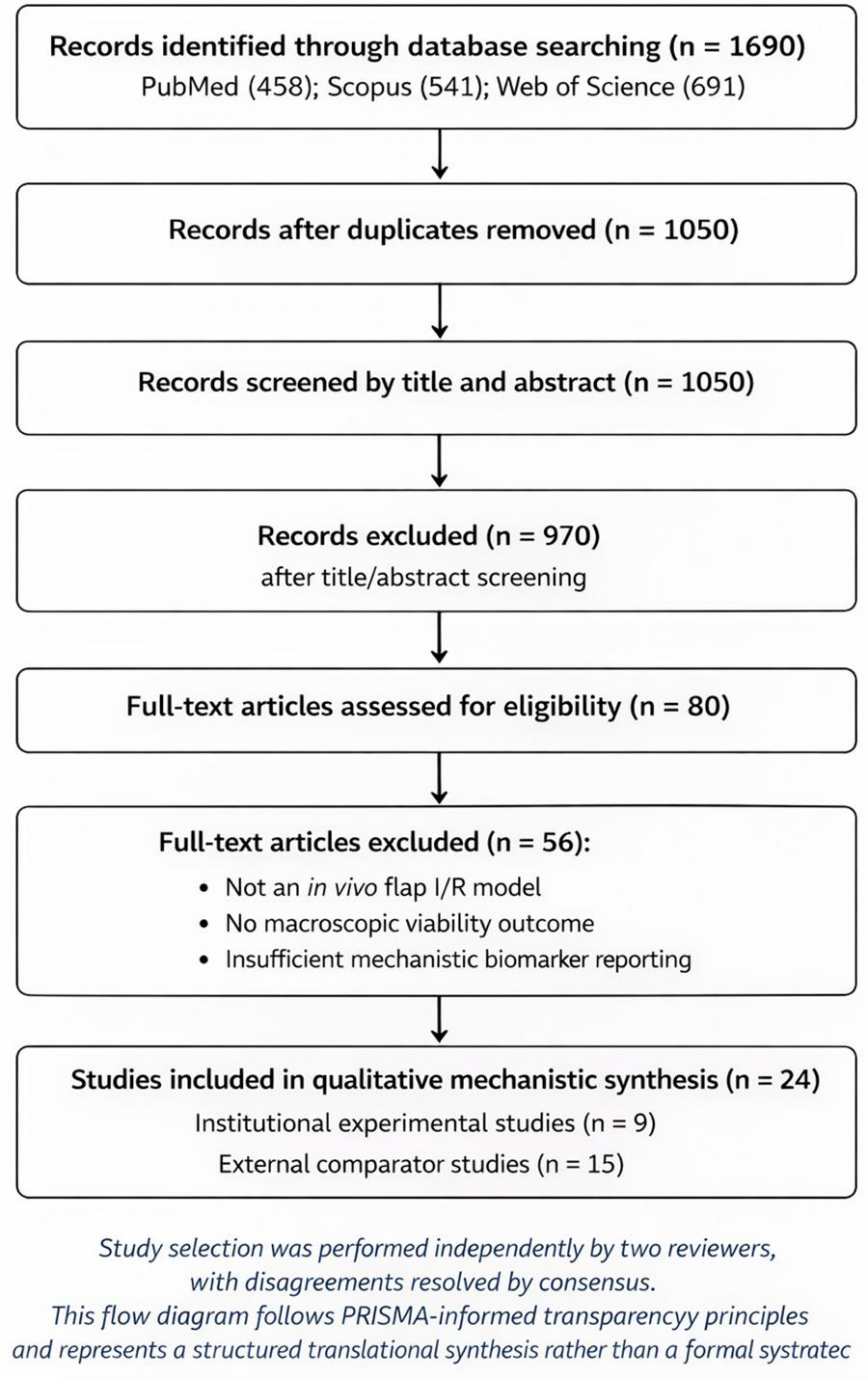
PRISMA-informed study selection flow diagram. This diagram illustrates the structured study identification and selection process following PRISMA-informed principles. It does not represent a formal systematic review flow diagram but is adapted to reflect a structured translational synthesis with predefined eligibility criteria. This flow diagram follows PRISMA-informed principles but reflects a structured mechanistic synthesis rather than a formal systematic review.

A total of 24 studies were included in the final structured mechanistic synthesis (9 institutional, 15 external comparator studies). A visual PRISMA flow diagram is provided in Figure 2.

Extracted variables included:

- Flap type/dimensions, ischemia duration, reperfusion duration, intervention type and timing, sample size.
- Macro-viability outcome (%, area, cm^2^).
- Oxidative markers (MDA, related indices), inflammatory markers (MPO, neutrophils, TNF-α), antioxidant status (GSH, SOD, CAT), NO-related endpoints, histology/scoring if available.

### Analytical approach (why no meta-analysis?)

Because heterogeneity was predefined and substantial (flap types, ischemia severity, intervention windows, outcome definitions, endpoint days), quantitative pooling risked false precision. Similar methodological cautions regarding heterogeneity and interpretability have been emphasized in experimental translational reviews (15, 16).

Therefore:

- Studies were stratified by model type and ischemia severity, with a specific focus on comparable severe ischemia epigastric island flap subgroups when relative effect estimation was biologically meaningful.
- A mechanistic convergence framework summarized directionality and cross-study reproducibility across four axes: lipid peroxidation, neutrophil activity, antioxidant restoration, and NO preservation/microvascular integrity.
- Where biologically comparable, survival changes were descriptively evaluated within severe ischemia subgroups to preserve interpretability.

### Definition of severe ischemia

Severe ischemia was operationally defined as ≥8 h of pedicle occlusion in axial island flap models, based on consistent transition from partial to extensive necrosis patterns reported across multiple experimental studies in the literature (16, 18)..

This threshold was used for biologically comparable subgroup interpretation rather than for statistical pooling.

### Translational framing

Mechanistic findings were interpreted relative to clinical analogs:

- Free flap thrombosis and re-exploration
- Reperfusion after revision/thrombectomy
- Prolonged warm ischemia in replantation
- Compromised beds (diabetic/irradiated)
- Congestion relief and microvascular dysfunction

External comparator studies were selected to represent key mechanistic clusters (oxidative modulation, anti-inflammatory signaling, metabolic stabilization, endothelial protection) identified in the institutional series (2, 3, 7–13).

### Ethics statement

This article is a structured narrative synthesis of previously published experimental studies. No new animal or human subjects were involved in the preparation of this work; therefore, additional institutional ethical approval was not required. All institutional experimental studies referenced herein were conducted under ethical approvals as reported in their original publications (7–13).

### AI-assisted visualization

Some schematic figures were created with the assistance of AI-based visualization tools for graphical rendering only. All scientific content, conceptual design, and interpretation were developed by the authors.

## Statistical analysis

Given the experimental nature of included studies and the heterogeneity of models, analyses were descriptive and mechanistic rather than inferential. When reported, original study *p*-values were preserved as published; no cross-study pooled effect sizes were generated (15).

Key heterogeneity domains precluding meta-analysis included:

- Model (muscle vs. random skin vs. epigastric island vs. venous)
- Ischemia duration (e.g., 2 h to 12 h)
- Endpoint day (acute biochemical sampling vs. day-7 necrosis vs. day-14 histology)
- Outcome metrics (%, cm^2^, NBT staining)
- Intervention timing (pre-ischemia, pre-reperfusion, postconditioning)

Within comparable severe-ischemia subgroups, effect direction and relative magnitude were descriptively contextualized against ischemic controls to preserve biological interpretability without generating artificial cross-model precision. No cross-study hypothesis testing was performed, and reported *p*-values reflect within-study comparisons only, thereby avoiding artificial statistical equivalence across biologically dissimilar models (15, 16).

Magnitude differences are presented descriptively within comparable model contexts and should not be interpreted as pooled superiority estimates across interventions. This approach was chosen to prioritize biological coherence over artificial statistical precision. The intent of this framework is hypothesis generation and mechanistic prioritization rather than quantitative interventional ranking.

## Results

The 24 included *in vivo* experimental studies—comprising 9 institutional investigations and 15 external comparator studies—are summarized in Tables 1, 2, respectively. These findings should be interpreted within model-specific experimental contexts rather than as direct cross-study comparisons.

**Table 1:** Institutional experimental flap ischemia–reperfusion studies (mechanistic synthesis) (*n* = 9 of 24 included studies) **.**

Study (author, year)	Model	Ischemia/endpoint	Intervention (timing)	Macro-viability outcome	Key mechanistic signal
Yan et al. (2010) (4)	Muscle flap	4 h/endpoint as reported	Postconditioning	Improved survival	Reduced oxidative stress; reduced edema; enhanced angiogenic signalingg
Yan et al. (2010) (5)	Venous flap	Conditioning/endpoint as reported	Pre-arterialization+delay	High survival (∼98%)	Enhanced microvascular remodeling
Akdemir et al. (2011) (7)	Muscle flap	4 h/endpoint as reported	Taurine	Survival increased	Reduced MDA; reduced neutrophil-mediated inflammation
Kerem et al. (2014) (8)	Random skin flap	I/R/day 7	Melatonin	Necrosis reduced	Reduced MDA; increased SOD and CAT activity
Akdemir et al. (2020) (12)	Epigastric flap	10 h/day 7	Trimetazidine	Viable area increased	Reduced MDA; reduced MPO; increased GSH; increased NO bioavailability
Seyid et al. (2021) (10)	Epigastric flap	6 h/day 7	Ceruloplasmin	Necrosis reduced	Reduced MDA; increased catalase activity
Akdemir et al. (2023)	Epigastric flap	10 h/day 7–10	Edaravone	No significant effect	No significant change in MDA, GSH, or NO
Eyuboglu et al. (2024) (11)	Epigastric flap	10 h/day 7–10	Propionyl-L-carnitine	Viable area increased	Reduced MDA; reduced MPO; increased GSH; increased NO bioavailability
Akdemir et al. (2025) (13)	Epigastric flap	10 h/day 7	Lutein	Survival increased	Reduced MDA; increased GSH; reduced MPO; increased NO bioavailability

Endpoint timing and outcome assessment methods are reported as described in the original studies. Due to heterogeneity in ischemia duration, endpoint timing, and measurement techniques, results are presented using a standardized qualitative–semiquantitative framework to ensure cross-study comparability while preserving data fidelity.

**Table 2:** External comparator experimental studies (*n* = 15) investigating pharmacologic or conditioning strategies.

Study (author, year)	Flap model	Ischemia duration	Endpoint	Survival effect	Key biomarkers	Primary mechanistic axis	Overall direction
Stadelmann et al. (1998) (19) [Aprotinin]	Ventral epigastric pedicled flap	10 h	Day 7	Improved survival (52% vs. 29%)	Reduced MPO	Neutrophil–protease inhibition	Protective
Muneuchi et al. (2013) (20) [D-allose]	Random dorsal flap	6 h venous clamp	Day 7	Improved survival	Reduced MDA; reduced MPO; increased SOD	ERK-mediated oxidative modulation	Protective
Niu et al. (2024) (21) [Dietary nitrate]	Random flap (diabetic)	Distal ischemia	Day 7	Increased survival area	Increased NO bioavailability; reduced ROS	Endothelial/NO preservation	Protective
Gideroglu et al. (2009) (22) [Montelukast]	Epigastric island flap	12 h	Day 7	Improved survival	Reduced MDA; reduced MPO; increased GSH	Leukotriene–neutrophil suppression	Protective
Eroglu et al. (2017) (23) [Propofol]	Epigastric island flap	2 h	Day 14	Improved survival	Reduced MDA; increased SOD	Anti-oxidative	Protective
Uysal et al. (2012) (24) [Dexmedetomidine]	Epigastric island flap	12 h	Day 7	Improved survival	Reduced MPO; reduced TNF-α	Anti-inflammatory	Protective
Kuroki et al. (2022) (31) [Ebselen]	Epigastric island flap	6 h	Day 7	Improved survival	Reduced lipid peroxidation	ROS scavenging	Protective
Aslan et al. (2014) (25) [EGCG]	Epigastric island flap	0–12 h subgroups	Day 7	Improved survival (9 h subgroup strongest)	Reduced MDA; increased SOD; reduced TNF-α	Polyphenol antioxidant/anti-inflammatory	Protective (severity-dependent)
Chen et al. (2018) (27) [Luteolin]	Random flap	6 h	Day 7	Improved survival	Reduced IL-6; reduced TNF-α	Anti-inflammatory flavonoid	Protective
Eskitascioglu et al. (2011) (26) [Lidocaine]	Epigastric flap	8 h	Day 7	Improved survival	Reduced MDA: no change in MPO	Membrane stabilization/partial inflammatory modulation	Protective
Sun et al. (2013) (28) [Isoflurane preconditioning]	Random flap	6 h	Day 7	Improved survival	Reduced IL-1β	Ischemic conditioning mimicry	Protective
Rezaeian et al. (2008) (29) [Erythropoietin]	Random flap	6 h	Day 7	Improved survival	Increased VEGF	Angiogenic signaling	Protective
Taleb et al. (2014) (32)[Metformin]	Random dorsal flap	6 h	Day 7	Improved survival	Reduced MDA; increased SOD	Antioxidant hormone	Protective
Knox et al. (1994) (34)[NOS inhibitors]	Random flap	6 h	Day 7	Improved survival	Reduced MPO; reduced ROS	Membrane stabilization	Protective
Hao et al. (2019) (30)[Hydrogen preconditioning]	Epigastric flap	3 h	72 h	No significant effect	Increased RIP1/RIP3	Necroptosis axis activation	Not protective

External comparator studies were selected to represent biologically relevant pharmacologic or conditioning strategies aligned with the mechanistic axes identified in the institutional dataset. Due to differences in flap physiology and vascular architecture, direct quantitative comparisons across models were not performed; instead, findings were interpreted within a shared mechanistic framework.

### Surgical conditioning strategies (upstream perfusion physiology)

- Postconditioning (muscle flap models): consistent reductions in oxidative injury markers (e.g., MDA) and edema with improved viability, supporting the concept that reperfusion choreography modulates downstream injury.
- Pre-arterialization+delay (venous flap): near-complete macro-viability within the respective experimental model.

### Pharmacologic modulation (mechanistic clusters)

#### Antioxidant/anti-inflammatory signature

Interventions in this cluster generally aligned with: MDA↓, MPO↓, antioxidant defenses↑, viability↑.

#### Metabolic modulators (mitochondrial/energetic stability)

In comparable severe ischemia epigastric island flap conditions, metabolic modulation showed consistent macro-viability benefits accompanied by oxidative/inflammatory attenuation and NO preservation:

In the severe 10-hour epigastric island flap model, metabolic modulators demonstrated improved survival relative to ischemic controls within their respective experimental contexts, accompanied by consistent reductions in lipid peroxidation and neutrophil activity with preservation of antioxidant defenses and nitric oxide bioavailability, as reported in the respective primary studies.

Where reported, statistical significance reflects within-study comparisons between treated and ischemic control groups as described in the original publications; no statistical testing was performed across studies.

#### Endogenous protective protein modulation

- Ceruloplasmin: marked necrosis reduction with catalase upregulation and MDA suppression, supporting a metal-catalyzed oxidative injury modulation component.

#### Negative outcome as mechanistic clarification (edaravone)

- Edaravone: under severe epigastric island I/R conditions, no meaningful macro-viability benefit and no consistent shift in core markers (MDA/GSH/NO), suggesting that isolated radical scavenging may be insufficient without parallel microvascular or inflammator*y* axis modulation.

### External comparator anchoring (examples)

External experimental studies reinforce convergence across models:

- Propofol improved viability and antioxidant markers with reduced MDA/oxidative stress in an epigastric island flap I/R model.
- Dexmedetomidine reduced NO/MDA/MPO and decreased necrosis in rat epigastric island flap I/R injury.
- Lidocaine improved survival and reduced inflammatory/oxidative injury markers after reperfusion injury.
- Epigallocatechin-3-gallate (EGCG) showed biochemical improvements and reduced necrosis particularly in prolonged ischemia subgroups in epigastric island flaps. (Table 3)

**Table 3:** Expanded mechanistic convergence matrix across experimental flap I/R models.

Mechanistic axis	Surgical conditioning	Antioxidant modulation	Inflammation modulation	Metabolic modulation	NO/endothelial	Angiogenic	Boundary example
Lipid peroxidation (MDA reduction)	✓	✓	±	✓	±	±	No consistent effect observed
Neutrophil activity (MPO reduction)	±	±	✓	✓	±	–	No consistent effect observed
Antioxidant restoration (SOD/GSH/CAT increase)	±	✓	±	✓	±	–	No consistent effect observed
NO bioavailability preservation	±	±	±	✓	✓	±	No consistent effect observed
Microvascular remodeling	✓✓	–	–	±	✓	✓	Consistent in conditioning-based models
Mitochondrial stabilization	–	±	–	✓✓	–	–	Metabolic-targeted interventions
Necroptosis modulation	–	–	–	–	–	–	Edaravone (no mechanistic effect)
Consistent macro-viability improvement	✓	✓	✓	✓	✓	±	Model-dependent effect

Legend: ✓ consistent effect across multiple models; ± variable or context-dependent effect; – not a primary mechanism; ↔ no significant effect; ✓✓ strong and reproducible effect across models; ✗ no consistent macro-viability improvement. This matrix reflects the interconnected nature of ischemia–reperfusion injury pathways, where mitochondrial dysfunction, oxidative stress, neutrophil activation, and endothelial nitric oxide dysregulation form a self-amplifying injury network rather than independent processes.

## Discussion

### A mechanistic convergence model across flap I/R paradigms

This 20-year institutional experimental series—spanning muscle flaps, random-pattern skin flaps, epigastric island flaps, and venous flap paradigms—supports a coherent and reproducible injury architecture in which reperfusion amplifies tissue loss beyond the primary ischemic insult. Institutional data were integrated with independent external experimental studies to provide mechanistic anchoring and enhance translational relevance. Although institutional studies are prominently represented due to protocol continuity, external comparator studies were systematically incorporated to minimize overrepresentation bias and to improve generalizability (7–13).

Across models, beneficial interventions consistently aligned with a shared biological signature: attenuation of lipid peroxidation, suppression of neutrophil-mediated inflammatory activity, restoration of endogenous antioxidant capacity, and preservation of endothelial function and nitric oxide (NO) bioavailability (7–13). This convergence suggests that translational success is more likely when interventions target multiple nodes of the oxidative stress–inflammation–microvascular dysfunction axis rather than a single downstream pathway. These pathways are not independent; mitochondrial dysfunction amplifies reactive oxygen species (ROS) generation, which in turn promotes neutrophil activation and endothelial nitric oxide dysregulation, creating a self-propagating injury loop.

Importantly, this mechanistic signature is not confined to institutional data. Independent external epigastric island flap I/R studies demonstrate closely overlapping profiles. For example, propofol administration significantly reduced necrosis area compared with ischemic controls and was associated with decreased oxidative stress (MDA), increased antioxidant capacity (SOD, CAT, TAC), and improved histopathological outcomes, including reduced edema, inflammation, and fibrosis—collectively mirroring the multi-axis protective profile observed across effective institutional interventions (23).

Different flap models represent distinct physiological and vascular conditions; therefore, findings are not intended to be directly interchangeable across models but should be interpreted within a shared mechanistic framework. This cross-model convergence supports the concept that reproducible biological signals, rather than isolated experimental findings, should guide translational prioritization in flap salvage strategies. To contextualize this heterogeneity, flap models exhibit inherent physiological differences with specific advantages and limitations. Muscle flaps demonstrate higher metabolic demand and lower ischemic tolerance, whereas random-pattern skin flaps provide reproducible ischemic gradients but lack axial vascular control. Epigastric island flaps offer a clinically relevant axial vascular model, while venous flaps represent unique perfusion dynamics distinct from arterialized systems. These differences limit direct comparability but collectively strengthen mechanistic generalizability across diverse ischemic conditions.

### Severity matters: why “comparable severe ischemia” is the most translational subgroup

A central interpretive strength of this synthesis is the stratified focus on biologically comparable severe ischemia subgroups rather than cross-model pooling. In the institutional dataset, the severe 10-hour epigastric island flap paradigm provides a stringent, clinically analogous stress test. Within this context, trimetazidine, propionyl-L-carnitine, and lutein demonstrated pronounced responses, whereas milder ischemia paradigms in the literature often showed attenuated or more variable macro-viability shifts (9, 11–13). Notably, several effective agents in the broader literature, including luteolin and EGCG, belong to the flavonoid/polyphenol family, supporting the idea that these compounds may modulate oxidative-inflammatory signaling beyond simple radical scavenging (25, 27).

External evidence supports severity dependence as a generalizable phenomenon. In an epigastric island flap model stratified across different ischemia intervals, EGCG produced consistent biochemical improvement and showed its clearest necrosis reduction signal particularly in the 9-hour subgroup, where ischemic burden crossed into a partial-necrosis range (25). This aligns well with the concept that therapeutic signal becomes most clinically relevant once endogenous defenses are overwhelmed, which is also the scenario most analogous to delayed free flap salvage (25).

Additional external comparators reinforce the concept of multi-axis protection. Ebselen improved flap survival and reduced tissue injury in a rat epigastric skin flap I/R model, supporting oxidative-pathway targeting beyond simple scavenging alone (31). Similarly, pharmacologic modulation through the nitric oxide system has been linked to improved flap outcomes. Metformin, for example, has been shown to enhance flap survival through nitric oxide–dependent mechanisms, with its protective effect largely abolished by NOS inhibition with L-NAME, underscoring the importance of endothelial nitric oxide signaling in microvascular preservation during reperfusion (32). Earlier work also demonstrated that iNOS-related signaling and broader NOS modulation can materially alter flap survival patterns, further emphasizing the central role of endothelial–microvascular regulation in ischemia–reperfusion injury (33, 34).

### Why metabolic modulation can outperform isolated scavenging under high ischemic burden

The pronounced viability improvements observed with trimetazidine and propionyl-L-carnitine are consistent with an upstream, mitochondria-centered protective logic (9, 11). Under prolonged ischemia, mitochondrial dysfunction and reperfusion-triggered ROS burst act as injury multipliers that propagate lipid peroxidation, inflammatory recruitment, and endothelial dysfunction. Agents that stabilize mitochondrial energetics and reduce ROS generation at its source may exert broader downstream effects than compounds that primarily scavenge radicals after inflammatory amplification and microvascular obstruction are already established (9, 11, 23, 26).

The external lidocaine study provides a useful comparator illustrating both overlap and nuance. Lidocaine significantly increased flap survival and suppressed MDA, yet did not consistently reduce MPO or neutrophil accumulation; the signal therefore appears more compatible with membrane stabilization and partial oxidative protection than with full-spectrum anti-inflammatory modulation (25). This reinforces a key translational point: macro-viability can improve through different mechanistic routes, but the most robust and reproducible protection in severe ischemia appears to involve broader multi-axis modulation (9, 11, 23–27, 31–34).

### Surgical conditioning: structural microvascular adaptation as upstream protection

Surgical conditioning strategies in the institutional series—particularly pre-arterialization plus delay in venous flap paradigms—produced near-complete macro-viability within the experimental context (5). Although surgical preconditioning strategies such as delay procedures and venous flap arterialization demonstrate near-complete survival in experimental settings, their clinical applicability may be limited by procedural complexity, additional operative time, and patient-specific vascular variability. This degree of protection is notable because conditioning strategies act upstream by reshaping perfusion physiology and microvascular architecture, promoting collateral recruitment and adaptive remodeling (5, 28, 29). From a translational standpoint, this supports a pragmatic hierarchy: first restore and optimize perfusion structurally, then consider pharmacologic adjuncts as biologic stabilizers that dampen oxidative and inflammatory amplification during reperfusion (5, 28, 29). Hydrogen preconditioning has also been investigated as a potential antioxidant strategy in experimental flap I/R models. However, a rat epigastric flap study demonstrated no meaningful improvement in survival despite modulation of necroptosis signaling, suggesting that isolated radical scavenging may be insufficient to overcome complex reperfusion injury pathways in certain experimental contexts (30).

### The “edaravone boundary condition”: why a negative result strengthens the framework

A major risk in the experimental flap I/R literature is the assumption that any antioxidant should work. In contrast, the institutional edaravone negative result under severe ischemia is better interpreted as a mechanistic boundary condition than as a weakness (12). In high-burden I/R, isolated radical scavenging may be insufficient if endothelial dysfunction, microvascular obstruction, and inflammatory amplification remain dominant drivers of secondary necrosis (12, 26, 31–34). This supports prioritizing multi-axis interventions—oxidative, inflammatory, and endothelial—over isolated scavengers when designing salvage-adjunct strategies (9, 11, 12, 23–27, 31, 32).

### Translational implications for free flap salvage

**Figure 3:**
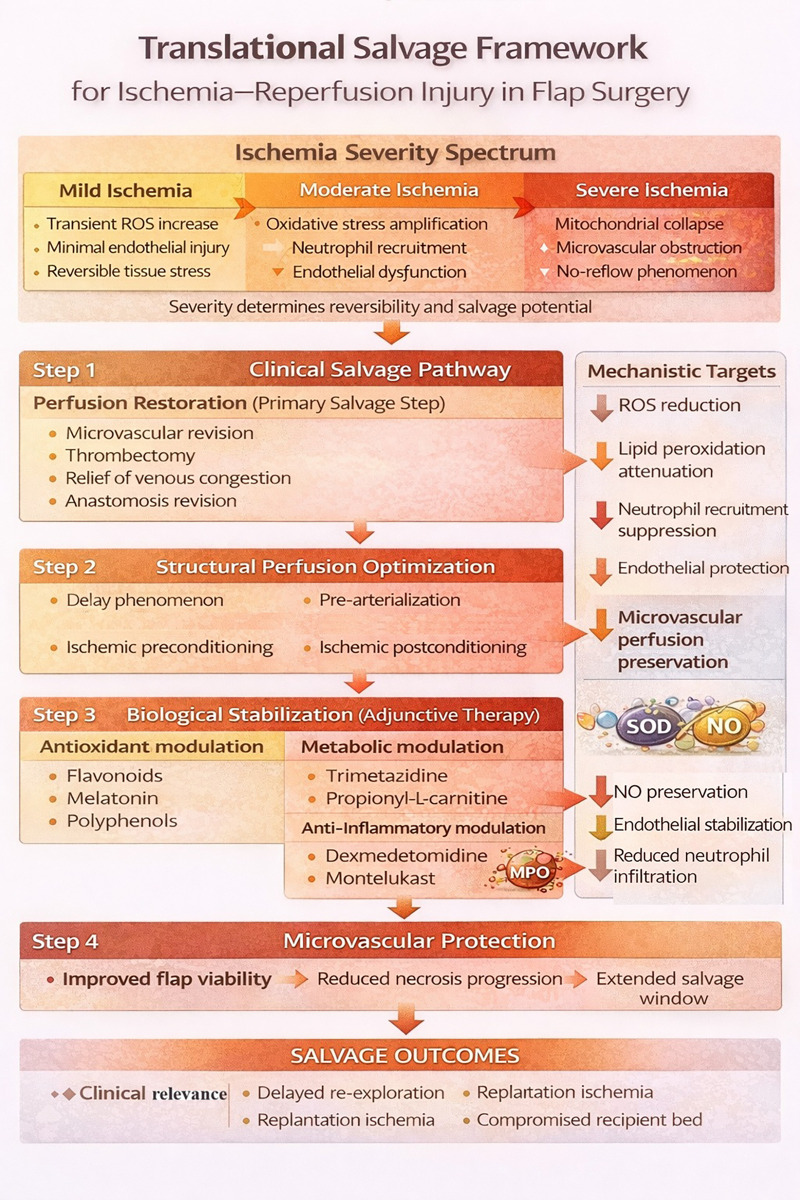
Translational salvage framework for ischemia–reperfusion injury in flap surgery. This framework integrates ischemia severity, surgical salvage strategies, and biologic modulation into a stepwise translational model. Severity-dependent injury progression determines reversibility and salvage potential, guiding a hierarchical approach: (1) restoration of perfusion, (2) structural optimization of microvascular flow, and (3) adjunctive biologic stabilization targeting oxidative stress, inflammation, and endothelial dysfunction. Multi-axis modulation is associated with improved flap viability and extended salvage potential under high ischemic burden.This schematic figure was created with the assistance of AI-based visualization tools for graphical rendering only. All scientific content and conceptual design were developed by the authors.

Clinically, free flap salvage most often occurs after arterial thrombosis with delayed re-exploration, venous congestion, or reperfusion after microvascular revision—scenarios that more closely resemble severe I/R stress than mild experimental paradigms (2, 3). The translational value of this synthesis is therefore not that specific percentage gains can be transferred directly into practice, but that a mechanistic prioritization emerges (Figure 3). The experimental–clinical correspondences that underpin this translational interpretation are summarized in Table 4. Effective adjuncts for salvage should plausibly: (1) attenuate lipid peroxidation, (2) limit neutrophil-driven inflammatory amplification, and (3) preserve endothelial/NO-dependent microvascular function during reperfusion (9, 11, 23–27, 31–34).

**Table 4:** Refined translational relevance framework for free flap salvage (mechanism-to-decision integration).

Experimental feature	Clinical analog	Translational message
Severe ischemia (6–12 h)	Delayed re-exploration after thrombosis	Adjunctive pharmacologic modulation is most relevant in high-burden ischemic states, where reperfusion injury contributes significantly to tissue loss.
NO preservation	Endothelial dysfunction after revision	Therapeutic strategies targeting endothelial function and nitric oxide bioavailability should be prioritized to restore microvascular perfusion.
MPO reduction	Post-reperfusion inflammatory surge	Modulation of neutrophil-driven inflammation may reduce secondary microvascular damage and improve flap viability.
Metabolic modulation	Prolonged warm ischemia in replantation	Mitochondrial-targeted therapies may extend the reversible ischemic window and improve cellular resilience.
Delay phenomenon	Staged reconstruction	Structural conditioning strategies provide robust protection by enhancing microvascular adaptation and tissue tolerance.
Necroptosis activation (hydrogen failure)	Irreversible reperfusion injury	Isolated antioxidant strategies may be insufficient once programmed cell death pathways are activated.
Multi-axis modulation	Borderline ischemic territory	Combined targeting of oxidative stress, inflammation, and endothelial dysfunction is likely required for effective salvage in high-risk scenarios.

This framework is intended to support clinical interpretation of experimental findings rather than to establish direct treatment recommendations. Translational inferences should be validated in prospective clinical studies.

Accordingly, future translational work should move away from isolated antioxidant screening and toward severity-stratified preclinical designs, standardized endpoint panels, and objective perfusion assessment. Given the substantial biological and methodological heterogeneity across studies (Table 5), quantitative meta-analysis was not considered appropriate. Instead, a structured mechanistic synthesis approach was adopted to preserve biological interpretability. In practical terms, perioperative adjunct development for salvage should be mechanistically guided and tested in models approximating delayed revision rather than low-stress ischemia windows (15, 16, 25, 31–34).

**Table 5:** Heterogeneity map and rationale for not performing meta-analysis.

Domain	Observed variability	Impact on quantitative synthesis
Flap type	Muscle/random skin/epigastric island/venous	Fundamentally different ischemic tolerance and microvascular architecture preclude direct biological comparability.
Ischemia duration	3–12 h (including diabetic distal ischemia)	Non-linear dose–response relationships prevent normalization across studies.
Endpoint timing	72 h – Day 14	Temporal heterogeneity introduces phase-dependent outcome variation, limiting cross-study alignment.
Outcome metrics	% necrosis/cm^2^ survival/NBT staining	Incompatible measurement scales prevent valid quantitative aggregation.
Intervention timing	Pre-ischemia/pre-reperfusion/postconditioning	Distinct therapeutic windows correspond to different mechanistic targets.
Marker panels	Partial vs. full oxidative/inflammatory profiling	Missing and non-uniform biomarker datasets preclude harmonized effect estimation.

Quantitative meta-analysis was not performed due to predefined biological and methodological heterogeneity across studies. Heterogeneity domains were defined *a priori* based on experimental design, outcome reporting, and mechanistic endpoints. A structured mechanistic synthesis approach with severity stratification was therefore adopted to preserve biological interpretability.

Although immediate microvascular revision remains the cornerstone of free flap salvage, biologically rational adjunctive modulation may help stabilize borderline ischemic territories during the reperfusion window and potentially extend the reversibility margin in selected high-risk scenarios (2, 3, 9, 11, 31, 32). This work should be interpreted as a hypothesis-generating translational framework rather than a comparative effectiveness analysis.

## Limitations

Several limitations should be acknowledged to appropriately bound interpretation. First, substantial heterogeneity across flap types (muscle vs. random-pattern skin vs. epigastric island vs. venous) and ischemia protocols precludes meaningful pooled inference; therefore, this work intentionally uses a stratified mechanistic synthesis rather than meta-analysis.

Second, most pharmacologic agents are represented by single primary studies within the institutional dataset, limiting replication certainty and raising the possibility of laboratory-specific protocol effects. Third, endpoint timing varies (acute biochemical sampling vs. day-7/10 macro-viability), whereas clinical salvage decisions often occur within hours of revision; thus, biochemical improvements do not automatically guarantee durable clinical benefit. Fourth, rodent models do not fully reproduce human free flap complexity, including larger perfusion territories, systemic inflammatory/coagulation interactions, comorbid physiology (diabetes, smoking, radiation), and heterogeneity of ischemic exposure. Finally, publication bias toward positive experimental findings cannot be excluded; however, inclusion and explicit interpretation of negative findings (e.g., edaravone) partially mitigates this concern by defining mechanistic boundary conditions.

Dose–response relationships and timing windows were not uniformly explored across agents, limiting mechanistic comparability and preventing robust inference regarding optimal dosing and administration schedules. Inter-study variability in necrosis quantification methods (planimetry, percentage area, NBT staining) further introduces measurement heterogeneity that constrains direct magnitude comparison across platforms. Variability in biomarker assessment methods and reporting thresholds across studies may further limit reproducibility and cross-study comparability. Accordingly, this work prioritizes mechanistic coherence over statistical aggregation, and magnitude differences should be interpreted within model-specific biological contexts rather than as direct interventional ranking. No claims of interventional superiority are made; instead, interventions are positioned within biologically comparable contexts to preserve translational coherence. The inclusion of multiple flap types introduces additional biological variability, which limits direct comparability but was necessary to capture shared mechanistic pathways. Finally, the absence of standardized reporting across experimental flap studies underscores the need for future consensus frameworks to improve comparability and translational alignment. This study is not designed as a comparative effectiveness analysis and does not support direct interventional ranking.Additionally, variability in biomarker assessment methods and reporting thresholds across studies may further limit reproducibility and cross-study comparability. Furthermore, alternative outcome systems—including apoptosis, necroptosis, and autophagy—were not uniformly assessed across studies, potentially limiting comprehensive mechanistic integration.

## Conclusion

Ischemia–reperfusion injury in flap surgery is governed by a reproducible oxidative stress–inflammation–microvascular dysfunction cascade. Across two decades of experimental investigation, interventions modulating multiple mechanistic axes demonstrated consistent protective patterns in severe ischemia contexts, whereas isolated single-target approaches showed limited reproducibility under high ischemic burden.

This translational synthesis provides a biologically coherent framework to guide rational adjunctive strategies in free flap salvage. Future progress will depend on standardized severe ischemia models, mechanistically guided combination strategies, objective perfusion metrics, and carefully designed early-phase clinical trials. Importantly, these findings should be interpreted as hypothesis-generating and require prospective validation in clinically aligned preclinical and early-phase human studies. While the present synthesis provides mechanistic coherence, direct clinical extrapolation requires cautious interpretation and prospective validation. This framework may serve as a conceptual bridge between experimental flap research and clinically actionable salvage protocols. However, these findings should be interpreted as a hypothesis-generating translational framework rather than definitive clinical guidance.
